# Immunogenicity Analysis of PCV3 Capsid Highly Expressed Using Baculovirus

**DOI:** 10.3390/ijms27114930

**Published:** 2026-05-29

**Authors:** Baoge Zhang, Lumen Chao, Yuchen Cai, Yufeng Li

**Affiliations:** Key Laboratory of Bacteriology, Ministry of Agriculture, College of Veterinary Medicine, Nanjing Agricultural University, Nanjing 210095, China; baogezhang@stu.njau.edu.cn (B.Z.); 2023107050@stu.njau.edu.cn (L.C.); 2024107045@stu.njau.edu.cn (Y.C.)

**Keywords:** PCV3, Cap, baculovirus expression system, VLPs, immunogenicity

## Abstract

Porcine circovirus type 3 (PCV3) capsid protein (Cap) is a key antigen for immunological studies and vaccine development. Different optimized PCV3 ORF2 sequences were used to construct six baculovirus transfer plasmids, with the pOET1.1-based design yielding the highest Cap level. Cap expression was confirmed by Western blot, IPMA and IFA. Recombinant baculovirus amplification was optimized, achieving the highest titer at an MOI of 0.1 with a 72 h harvest to 10^7.5^TCID_50_/0.1 mL, while maximal Cap production was obtained at an MOI of 0.1 with a 96 h harvest. PCV3 Cap virus-like particles (VLPs) were purified by sucrose density-gradient ultracentrifugation and cation-exchange chromatography, and TEM revealed spherical particles of approximately 17–20 nm. In mice, VLP immunization induced increasing antigen-specific IgG from day 14. Immunization increased both IgG1 and IgG2a without a significant difference, and post-immunization serum specifically recognized PCV3-positive passaged PK-15 cells in an indirect immunofluorescence assay. In splenic lymphocytes, IFN-γ, TNF-α, IL-4, and IL-10 mRNA levels were significantly upregulated (*p* < 0.01). Moreover, pig challenge data supported the protective potential of PCV3 Cap VLPs in the natural host. In our study, Cap assembled into VLPs and induced immune responses, providing a basis for PCV3 subunit vaccine development.

## 1. Introduction

PCV3 has been widely detected in the swine industry and is associated with diverse clinical conditions, including reproductive disorders and systemic inflammatory syndromes [1]. However, the pathogenesis and immune responses associated with PCV3 infection remain elusive, and highly effective antigen production platforms are still needed to support serological assays, systemic immunology studies, and vaccine evaluation. The capsid protein, encoded by ORF2, is the major structural and immunodominant antigen of circoviruses and the primary target of host antibody responses, making it a target for PCV3 antibody detection and an attractive immunogen for subunit or particle-based vaccine development [2,3,4,5]. Notably, Cap can self-assemble into VLPs that mimic native virion architecture while lacking viral genetic material, providing a safe and immunogenic antigen format for downstream applications [6,7,8].

A baculovirus-expressed full-length PCV3 Cap was reported as a high-quality coating antigen for an indirect ELISA to detect PCV3 IgG, supporting that insect-cell-derived Cap can be used as diagnostically relevant antigen [9]. In addition, Sf9-expressed PCV3 Cap was shown to self-assemble into VLPs and enable VLP-based serological detection [2]. A recombinant PCV2 ORF2 subunit vaccine has shown efficacy against PCV2 infection in pigs, demonstrating that Cap-based antigens can confer meaningful protection in the natural host [10,11,12,13,14,15]. More recently, a recombinant PCV2d baculovirus-derived capsid vaccine was reported to confer complete protection against PCV2d challenge in pigs, reinforcing VLP strategies as potent immunogen formats for circoviruses [16]. In parallel, official product documentation for licensed PCV2 vaccines describes baculovirus-engineered ORF2 antigen production, supporting the practical application of insect-cell-derived circovirus antigens in veterinary vaccination.

The insect cell-baculovirus platform has proven commercial translatability, including licensed products. Regulatory documentation for Cervarix specifies that HPV L1 proteins are produced using a recombinant baculovirus expression system in insect cells and assembled as VLPs during manufacture [17,18]. FluBlok is a licensed recombinant influenza vaccine produced by expressing hemagglutinin in insect cells using baculovirus, demonstrating scalable production of recombinant viral antigens by this platform [19,20]. Maltose-binding protein (MBP) has been widely used as a fusion partner to enhance recombinant protein expression and solubility, whereas foot-and-mouth disease virus 2A (F2A) peptides are commonly employed to enable co-expression of two proteins as translationally separated products from a single open reading frame [21,22]. For PCV3 Cap, previous studies have shown that the N-terminal arginine-rich NLS mediates nuclear targeting and can also reduce efficient recombinant expression; accordingly, deletion of this region may improve the recoverable expression of Cap [5]. In addition, a second putative NLS has been suggested in the middle region of PCV3 Cap, which may explain why residual nuclear localization can still be observed after N-terminal NLS deletion [3].

In this study, we constructed and compared six baculovirus expression vector system (BEVS) transfer plasmids encoding PCV3 Cap and derived variants. The Cap expression in Sf9 cells was optimized using baculovirus proliferation and Cap expression conditions. PCV3 Cap VLPs were purified by sucrose density-gradient ultracentrifugation combined with cation-exchange chromatography, and its humoral and cytokine responses were evaluated by mice immunization. Furthermore, the protective efficacy of the VLPs was evaluated in the natural host.

## 2. Results

### 2.1. Construction of Six Recombinant PCV3 Cap Plasmids

**Figure 1:**
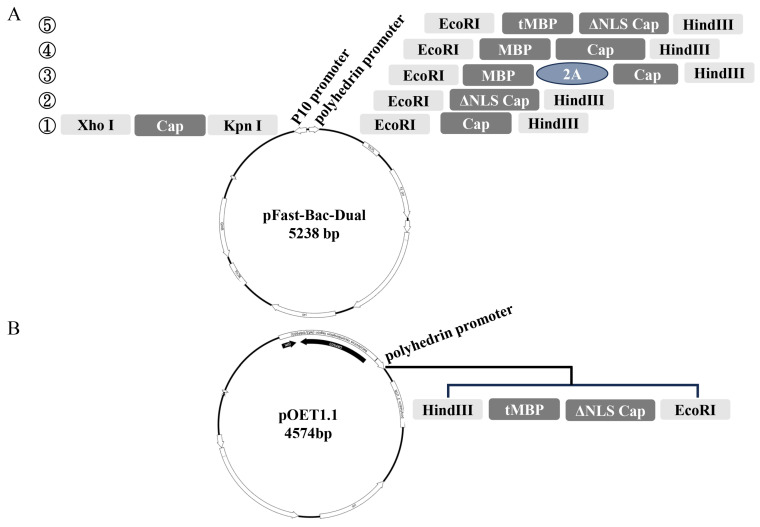
Schematic diagrams of baculovirus transfer plasmids encoding PCV3 Cap and engineered variants. (**A**) The PCV3 ORF2 (Cap) sequence (GenBank: PP196470.1) was used to generate five recombinant plasmids in the pFastBac-Dual vector, including dual-copy Cap, ΔNLS Cap, M2ACap, MBP-Cap, and tMCap. The dual-copy Cap construct contained the full-length Cap sequence under both the p10 and polyhedrin (pH) promoters. The ΔNLS Cap construct was generated by deleting 32 amino acids from the NLS-containing region. M2A Cap was generated by fusing full-length MBP to the N-terminus of full-length Cap and inserting an F2A peptide between MBP and Cap. MBP-Cap contained a direct fusion of full-length MBP to the N-terminus of full-length Cap. tMCap was generated by replacing the N-terminal 32 amino acids of Cap with the N-terminal 42 amino acids of MBP. (**B**) The tMCap expression cassette was further subcloned into pOET1.1 to generate the sixth construct, designated pOET1.1-based tMCap, for subsequent expression analyses.

PCV3 ORF2 based on GenBank accession no. PP196470.1 was used to generate six baculovirus transfer plasmids, designated dual-copy Cap, ΔNLS Cap, M2A Cap, MBP-Cap, tMCap, and pOET1.1-based tMCap (Figure 1).

### 2.2. Comparative Expression Analysis of Six PCV3 Cap Constructs in Sf9 Cells

**Figure 2:**
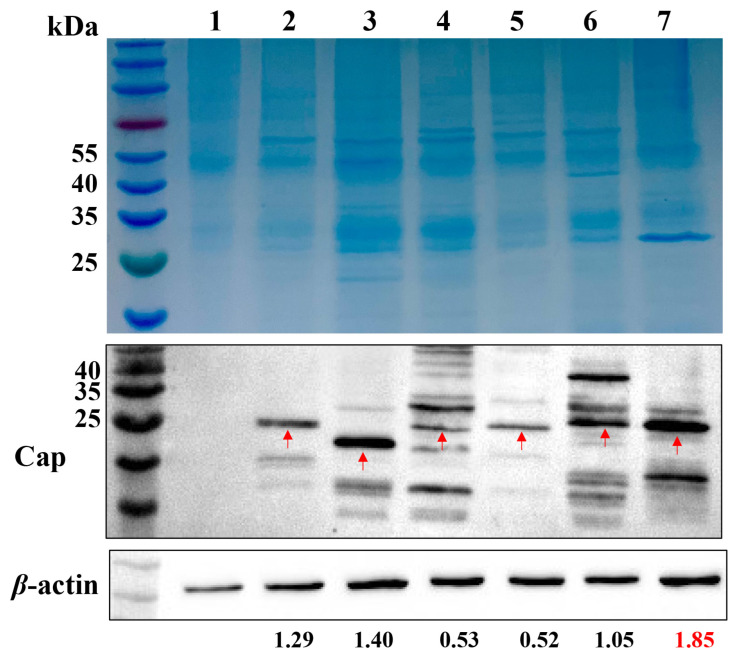
Screening of PCV3 Cap expression from six engineered constructs in Sf9 cells by SDS-PAGE and Western blot. Whole-cell lysates were separated by SDS-PAGE and visualized by Coomassie brilliant blue staining. Cap protein was detected using an anti-PCV3 Cap antibody, and β-actin served as the loading control. Samples are shown from 1–7 as follows: uninfected Sf9 cells, dual-copy Cap, ΔNLS Cap, M2A Cap, MBP-Cap, tMCap, and pOET1.1-based tMCap. The arrows indicate the target Cap protein bands. The corresponding Cap/β-actin ratios for the six recombinant constructs were 1.29, 1.40, 1.05, 0.53, 0.52, and 1.85, respectively. The red arrows indicate the target Cap protein bands identified in each recombinant construct and used for densitometric analysis. The red value indicates the condition with the highest relative Cap expression level.

To identify the most suitable construct for downstream VLP production, the expression of the six recombinant plasmids was evaluated in Sf9 cells by SDS-PAGE and Western blot (Figure 2). No Cap-specific band was detected in uninfected Sf9 cells, whereas all six recombinant constructs produced detectable Cap signals with clear differences in abundance. During the preliminary construct-screening stage, the expression level of Cap was relatively low in some constructs. Therefore, longer exposure was required to detect the target Cap band, which also resulted in the visualization of some non-specific bands. Densitometric analysis normalized to β-actin showed Cap/β-actin ratios of 1.29 for dual-copy Cap, 1.40 for ΔNLS Cap, 1.05 for M2A Cap, 0.53 for MBP-Cap, 0.52 for tMCap, and 1.85 for pOET1.1-based tMCap. In the M2A Cap construct, the F2A peptide did not appear to completely separate MBP from Cap, and multiple additional protein bands were observed, suggesting incomplete cleavage and the presence of heterogeneous products, which may also be related to partial instability of the recombinant protein under the experimental conditions used. In the MBP-Cap construct, no clear intact MBP-Cap fusion band was detected, suggesting that MBP and Cap were not efficiently expressed as a single fused protein. Among these constructs, pOET1.1-based tMCap exhibited the highest expression level and was therefore selected for subsequent experiments.

### 2.3. Verification of PCV3 Cap Expression in Sf9 Cells by Western Blot and Immunostaining

**Figure 3:**
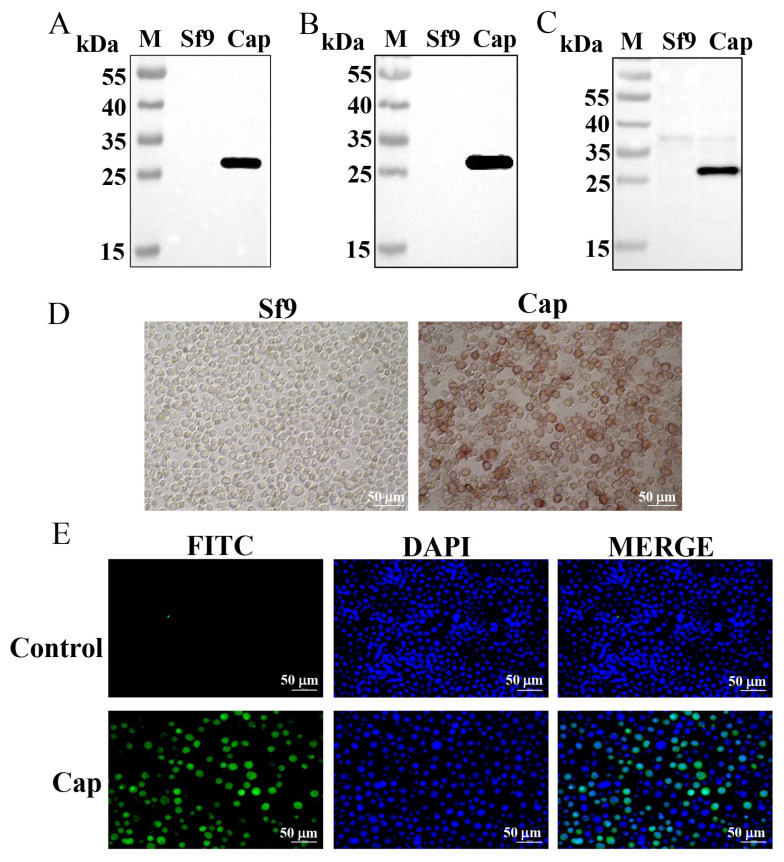
Confirmation of PCV3 Cap expression in Sf9 cells by Western blot, IPMA, and IFA. (**A**–**C**) Western blot of Sf9 cell lysates shows Cap-specific bands detected with an anti-His monoclonal antibody, a PCV3-Cap monoclonal antibody, and PCV3-positive porcine serum; uninfected Sf9 cells serve as the negative control. (**D**) IPMA detection of Sf9 cells infected with recombinant baculovirus was visualized using AEC substrate, showing red signals in both cytoplasm and nuclei of infected cells, whereas uninfected cells exhibited no obvious staining. (**E**) Indirect immunofluorescence shows Cap-specific staining in infected cells (green) with DAPI nuclear counterstaining (blue); merged images are shown, and the uninfected control displays minimal background.

Following construct screening, the pOET1.1-based tMCap design was used for expression validation in Sf9 cells. Western blot analysis using an anti-His monoclonal antibody, a PCV3-Cap monoclonal antibody, and PCV3-positive porcine serum consistently detected a distinct Cap-specific band in recombinant samples, whereas no corresponding signal was observed in uninfected Sf9 cells (Figure 3A–C). In parallel, an IPMA was performed to examine recombinant baculovirus infection in Sf9 cells (Figure 3D). AEC-based color development revealed clear red signals in both the cytoplasm and nuclei of infected cells, while the uninfected control group showed no obvious staining. In addition, IFA showed a strong Cap-specific green fluorescence signal in infected Sf9 cells, whereas the uninfected control exhibited minimal background; nuclei were counterstained with DAPI, and merged images further confirmed the specific staining pattern (Figure 3E). Collectively, these assays confirmed successful expression of PCV3 Cap in Sf9 cells.

### 2.4. Optimization of Recombinant Baculovirus Amplification Conditions

To maximize recombinant baculovirus titer and improve infection efficiency, amplification conditions were optimized by varying the multiplicity of infection and harvest time, and viral growth under each condition was evaluated. The highest titer was obtained at an MOI of 0.1 with a 72 h post-infection harvest, reaching 10^7.5^TCID_50_/0.1 mL. This condition was therefore selected as the optimal amplification strategy for subsequent experiments.

### 2.5. Optimization of Cap Protein Expression Conditions

**Figure 4:**
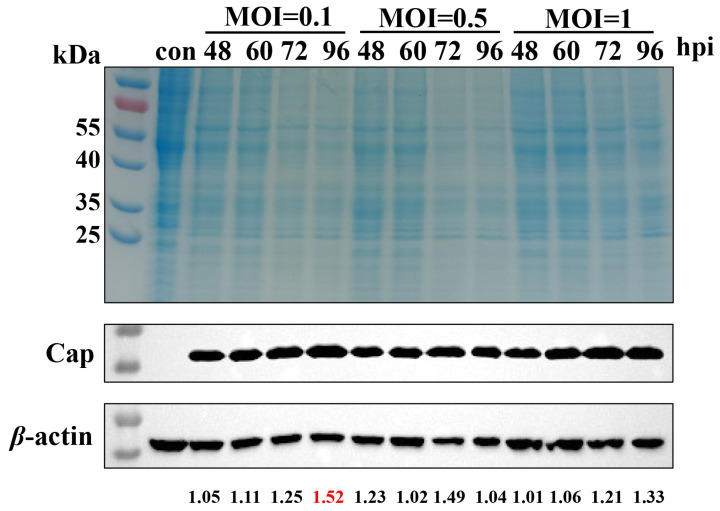
Optimization of PCV3 Cap protein expression in Sf9 cells under different MOI and infection-time conditions. Sf9 cells were infected with recombinant baculovirus at the indicated multiplicities of infection and harvested at different time points. Whole-cell lysates were analyzed by SDS-PAGE with Coomassie brilliant blue staining and by Western blot for Cap. β-actin was used as the loading control. Cap expression levels were evaluated by densitometric analysis normalized to β-actin, and the highest expression was observed at an MOI of 0.1 with a 96 h post-infection harvest. The red value indicates the condition with the highest relative Cap expression level.

Cap protein expression in Sf9 cells varied under different multiplicity of infection and harvest-time settings (Figure 4). When an MOI of 0.1 was used, Cap expression increased with prolonged infection time and reached the highest level at 96 h post-infection, as determined by Western blot analysis with β-actin as the loading control and densitometric normalization of Cap to β-actin. Based on these results, an MOI of 0.1 with a 96 h harvest was selected as the optimal condition for Cap protein production.

### 2.6. Purification and Characterization of PCV3 Cap VLPs

**Figure 5:**
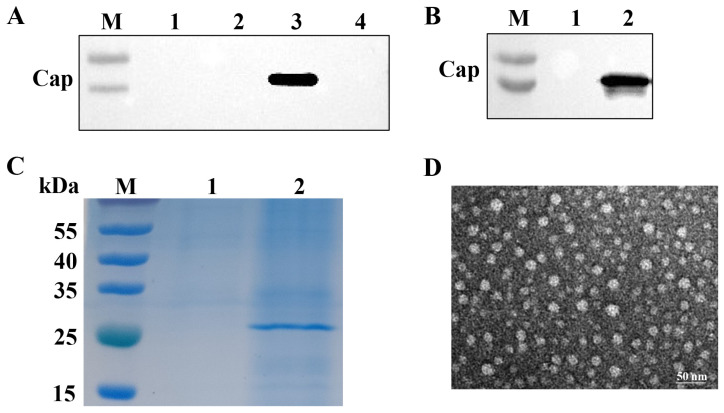
Purification and TEM characterization of PCV3 Cap VLPs. (**A**) Cap VLPs were separated by sucrose density-gradient ultracentrifugation and analyzed by Western blot, showing predominant enrichment in the 30–40% sucrose fraction. Lanes 1–4 represent the Sf9 cell negative control, 20–30% sucrose fraction, 30–40% sucrose fraction, and 40–50% sucrose fraction, respectively. (**B**) The 30–40% fraction was further purified by cation-exchange chromatography at pH 7.8, and the target product was efficiently eluted with 400 mM NaCl as confirmed by Western blot using a PCV3-Cap monoclonal antibody. Lanes 1 and 2 represent the 1 M NaCl eluate and 400 mM NaCl eluate, respectively. (**C**) SDS-PAGE with Coomassie staining shows a single major band at approximately 27 kDa after cation-exchange purification. Lanes 1 and 2 represent the 1 M NaCl eluate and 400 mM NaCl eluate, respectively. (**D**) Transmission electron microscopy reveals assembled VLPs with diameters of approximately 17–20 nm.

PCV3 Cap VLPs were purified using sucrose density-gradient ultracentrifugation followed by cation-exchange chromatography. After density-gradient separation with stepwise sucrose layers, Western blot analysis showed that Cap-containing VLPs were predominantly enriched in the 30–40% sucrose fraction (Figure 5A). Because the predicted isoelectric point of Cap was 10.84 and the purification buffer was maintained at pH 7.8, the 30–40% fraction was further processed by cation-exchange chromatography to remove residual host proteins and improve purity. Western blot and SDS-PAGE indicated that elution with 400 mM NaCl efficiently recovered the target product, yielding a prominent single band at approximately 27 kDa that could be specifically recognized by a PCV3-Cap monoclonal antibody (Figure 5B,C). Transmission electron microscopy performed by Zhenjiang Zhuanbo Testing Technology Co., Ltd. (Zhenjiang, China), revealed uniform, spherical particles with an estimated diameter of 17–20 nm, consistent with the expected size of PCV3 VLPs (Figure 5D). Collectively, these data demonstrate successful purification of PCV3 Cap VLPs with high purity and correct particle assembly.

### 2.7. Immunogenicity Analysis of PCV3 Cap VLPs

**Figure 6:**
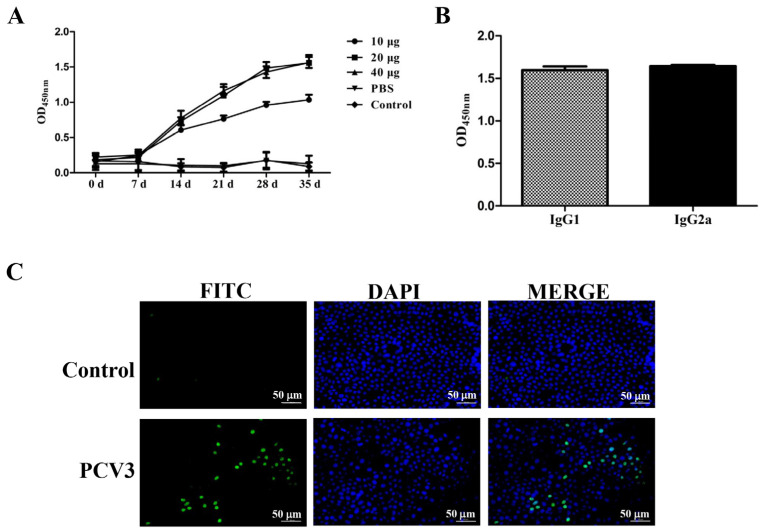
Humoral responses elicited by PCV3 Cap VLP immunization. (**A**) VLP-specific IgG kinetics were determined by indirect ELISA for mice immunized with 10, 20, or 40 μg PCV3 Cap VLPs, with PBS and control groups included as negative controls; antibody responses became detectable from day 14 and increased over time in immunized groups. (**B**) IgG subclass responses were assessed by ELISA, showing elevated IgG1 and IgG2a levels after immunization with no significant difference between subclasses. (**C**) Recognition of PCV3-positive passaged PK-15 cells by post-immunization serum was further evaluated by indirect immunofluorescence assay, showing specific fluorescence signals in virus-positive cells, whereas no specific signal was detected in the control group.

PCV3 Cap VLP-specific antibody responses were monitored by indirect ELISA in mice immunized with 10, 20, and 40 μg antigen. VLP-specific IgG was detected from day 14 post-immunization and increased steadily thereafter in all immunized groups. The 20 μg and 40 μg groups exhibited comparable antibody levels throughout the experimental period, and both groups consistently showed higher responses than the 10 μg group. In contrast, no detectable VLP-specific antibody response was observed in the PBS group or the control group (Figure 6A). To further characterize the IgG subclass profile elicited by PCV3 Cap VLPs, IgG1 and IgG2a levels were measured by ELISA using serum collected from the 20 μg immunization group. Both IgG1 and IgG2a were elevated following immunization, with IgG2a showing a slightly higher level than IgG1; however, the difference between the two subclasses was not statistically significant (Figure 6B). To further verify whether antibodies induced by PCV3-Cap VLP immunization could recognize PCV3-associated antigens, an indirect immunofluorescence assay was performed using post-immunization serum from VLP-immunized mice as the primary antibody and PK-15 cells persistently carrying PCV3 through serial passage as the target cells. The results showed that the immune serum specifically recognized PCV3-associated fluorescence signals in virus-positive cells, whereas no specific signal was observed in the control group (Figure 6C). These findings further demonstrate that antibodies induced by PCV3 Cap VLP immunization can recognize PCV3-related antigens in virus-positive cells (Figure 6C).

### 2.8. Cytokine Responses After Immunization

**Figure 7:**
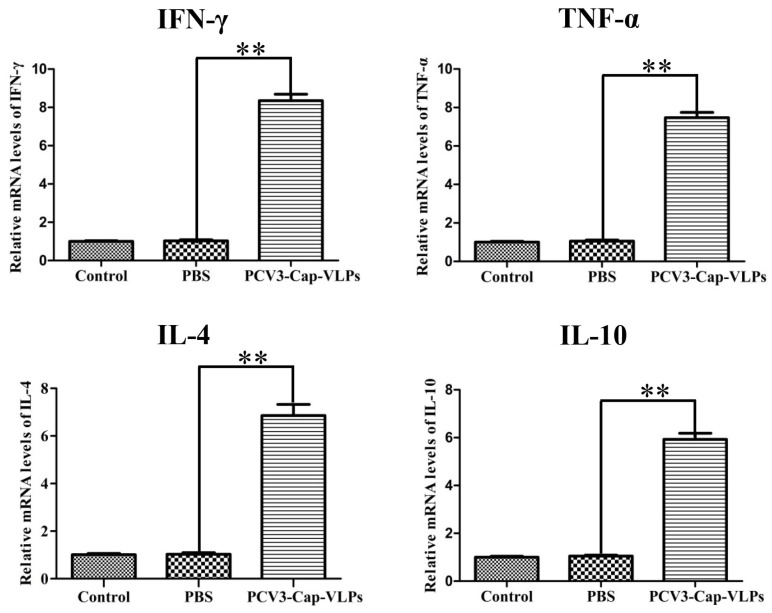
Cytokine mRNA responses in splenic lymphocytes after PCV3 Cap VLP immunization. Relative mRNA expression levels of IFN-γ, TNF-α, IL-4, and IL-10 were measured in mouse splenic lymphocytes after immunization with PCV3 Cap VLPs and compared with the control group. All four cytokine transcripts were significantly increased in the immunized group. All data are presented as the means ± SDs. ** *p* < 0.01.

To evaluate cytokine-associated transcriptional responses induced by PCV3 Cap VLP immunization, the relative mRNA expression levels of IFN-γ, TNF-α, IL-4, and IL-10 were measured in splenic lymphocytes. Compared with the control group, all four cytokine transcripts were significantly upregulated in the immunized group, showing highly significant differences with *p* < 0.01 (Figure 7).

### 2.9. Protective Efficacy of PCV3 Cap VLPs Immunization in Pigs

To assess the protective efficacy of PCV3 Cap VLPs in the natural host, pigs were subjected to immunization followed by PCV3 challenge, and the positive detection rate of serum viral load was determined at different time points after challenge. As shown in Table 1, no positive serum viral load was detected in the negative control group throughout the experiment. In the challenge control group, all pigs were positive from 3 dpi to 28 dpi. In contrast, the PCV3 Cap VLPs-immunized group showed a lower positive detection rate, with 2/5 pigs positive at 3 dpi and only 1/5 pigs positive from 7 to 28 dpi (Table 1). These results indicate that PCV3 Cap VLPs immunization reduced the positive detection rate of serum viral load after challenge and may confer partial protection in pigs.

## 3. Discussion

The capsid protein, encoded by ORF2, is the major structural and immunodominant antigen of PCV3 [23]. Cap-derived virus-like particles can mimic native-like epitopes without carrying viral genomes, making them suitable for immunogenicity evaluation and antigen development [3,24,25,26,27,28]. In this study, Cap expression in Sf9 cells was highly dependent on construct design and vector context. Among the six engineered constructs, the pOET1.1-based tMCap produced the highest Cap level by densitometry. These findings indicate that rational engineering of the Cap expression cassette is critical for improving antigen yield and may facilitate the development of standardized PCV3 VLP-based reagents for both diagnostic and vaccine-related applications.

Optimization of process parameters showed that the best conditions for virus amplification and protein production were not the same. The highest baculovirus titer was obtained at MOI 0.1 with a 72 h harvest, while Cap accumulation peaked at MOI 0.1 with a 96 h harvest. For purification, Cap-containing particles were enriched mainly in the 30–40% sucrose fraction and further purified by cation-exchange chromatography at pH 7.8, guided by the basic predicted pI of Cap [29,30,31]. The purified preparation yielded a dominant 27 kDa band recognized by a PCV3-Cap monoclonal antibody, supporting effective enrichment and antigen integrity. However, the present study was not designed as a full bioprocess characterization analysis, and further quantitative assessment of yield, purity, recovery, and reproducibility will be required to more fully evaluate the production performance of this workflow. From an application perspective, the purified PCV3-Cap VLPs may serve not only as standardized antigens for serological assays, but also as candidate immunogens for vaccine development. Given the frequent co-circulation of PCV2 and PCV3 in swine herds, these VLPs may also have potential value for future multivalent or combination vaccine design together with existing PCV2 vaccine strategies.

Purified VLPs were confirmed by TEM as uniform particles of approximately 17–20 nm, consistent with circovirus-like morphology. In mice, VLP immunization induced antigen-specific IgG from day 14, with 20 and 40 μg producing similar responses and both exceeding 10 μg, while controls remained negative. Immunization increased both IgG1 and IgG2a, with no significant difference between the two subclasses. In addition, post-immunization serum specifically recognized PCV3-positive passaged PK-15 cells in an indirect immunofluorescence assay, further supporting the antigenic relevance of PCV3 Cap VLPs and the biological specificity of the induced humoral response. Moreover, the mRNA levels of IFN-γ, TNF-α, IL-4, and IL-10 were significantly elevated in splenic lymphocytes, indicating cytokine-associated transcriptional activation after immunization. Importantly, the pig immunization–challenge experiment provided preliminary support for the biological relevance of PCV3 Cap VLPs in the natural host and suggests their potential for further evaluation in PCV3-related prevention and control. At the same time, several limitations should be acknowledged. Although the murine model is useful for preliminary immunogenicity evaluation, it cannot fully reflect the immune characteristics, disease progression, or protective efficacy of PCV3 infection in pigs. In addition, cytokine responses in this study were assessed only at the mRNA level, which reflects transcriptional activation but does not directly indicate cytokine secretion at the protein level.

In summary, this study establishes an optimized BEVS-based workflow for producing structurally intact PCV3 Cap VLPs, demonstrates their immunogenicity in mice, and provides preliminary support for their protective potential in pigs through the immunization–challenge experiment, highlighting their potential as standardized antigens for PCV3 serology and as candidate immunogens for further vaccine evaluation.

## 4. Materials and Methods

### 4.1. Design and Construction of Six Baculovirus Transfer Plasmids Encoding PCV3 Cap Variants

The PCV3 ORF2 sequence based on GenBank accession no. PP196470.1 was used to generate six recombinant plasmids. In all constructs, a C-terminal 6× His tag was introduced to facilitate downstream detection and purification. First, the Cap coding sequence was cloned into the pFastBac-Dual vector under both the p10 and polyhedrin (pH) promoters, with Xho I/Kpn I and EcoR I/Hind III restriction sites introduced into the two expression cassettes, respectively; this construct was designated dual-copy Cap. Second, an ΔNLS Cap was generated by deleting 32 amino acids from the NLS-containing region and cloning the modified sequence into the pH cassette of pFastBac-Dual using EcoR I/Hind III. Third, a construct designated M2A Cap was generated by fusing full-length MBP to the N-terminus of full-length Cap and inserting a F2A between MBP and Cap, followed by cloning into pFastBac-Dual using EcoR I/Hind III. Fourth, a full-length MBP-Cap fusion construct was generated by directly fusing MBP to the N-terminus of full-length Cap and cloning it into pFastBac-Dual using EcoR I/Hind III. Fifth, a mutant construct designated tMCap was generated by replacing the N-terminal 32 amino acids of Cap with the N-terminal 42 amino acids of MBP and cloning the resulting sequence into pFastBac-Dual. Finally, the tMCap sequence was subcloned into the pOET1.1 vector to generate the sixth construct. Plasmid synthesis and cloning were performed by Shanghai Generay Biotech Co., Ltd. (Shanghai, China). Based on the comparative expression analysis, the sixth construct, namely tMCap subcloned into pOET1.1, exhibited the highest protein expression level and was therefore selected for subsequent experiments. The monoclonal antibody against PCV3 Cap used in this study was generated previously in our laboratory [32].

### 4.2. Bacmid Generation Using the Bac-to-Bac System

Recombinant pFastBac Dual plasmids were transformed into DH10Bac competent *E. coli* to generate recombinant bacmids by site-specific transposition, followed by blue–white screening on triple-antibiotic LB agar containing kanamycin (50 μg/mL), tetracycline (10 μg/mL), and gentamicin (7 μg/mL) with X-gal/IPTG to select positive white colonies. Recombinant bacmid DNA was then transfected into Sf9 cells seeded at 1.5 × 10^6^ cells per well in 6-well plates using BaculoFECTIN II (Oxford Expression Technologies, Oxford, UK) at 1 μL reagent per 1 μg bacmid DNA. Cells were incubated at 28 °C and culture supernatants were harvested at 72–96 h upon appearance of typical cytopathic effects to obtain the P1 virus stock.

### 4.3. Amplification of Recombinant Baculovirus and Optimization of Culture Conditions

The P1 virus stock was used to infect Sf9 cells for further amplification. To obtain recombinant baculovirus with higher titer, amplification was performed under different multiplicities of infection and harvest time. Viral titers were determined by a TCID_50_ assay. Virus samples were serially diluted in 10-fold steps and inoculated onto Sf9 cells with five replicate wells per dilution. Positive wells were recorded, and TCID_50_ values were calculated using the Reed–Muench method [33].

### 4.4. Cap Expression and Immunostaining in Sf9 Cells

Sf9 cells were infected with recombinant baculovirus and collected at the time when typical cytopathic effects, including cell enlargement, rounding, and increased cell detachment, became evident. Cells were resuspended with culture supernatant, centrifuged at 300× *g* for 5 min, and the pellet was lysed in lysis buffer containing PMSF; clarified lysates were obtained by centrifugation at 10,000× *g* for 5 min, mixed with loading buffer, heat-denatured, and analyzed by SDS-PAGE and Western blot using PCV3-positive porcine serum, and a PCV3-Cap monoclonal antibody. Cap production was optimized by testing different MOIs and harvest time based on Cap band intensity. For immunostaining, infected Sf9 cells were examined by IPMA after fixation with methanol containing 1% H_2_O_2_ and staining with PCV3-Cap monoclonal antibody (1:1000) followed by HRP-conjugated goat anti-mouse antibody (1:5000) and AEC development, and by indirect immunofluorescence after 4% paraformaldehyde fixation using the same primary antibody with an FITC-conjugated secondary antibody and DAPI nuclear counterstaining.

### 4.5. Purification of PCV3 Cap VLPs by Sucrose Density-Gradient Ultracentrifugation

Sucrose solutions at 20%, 30%, 40%, and 50% (*w*/*v*) were prepared in sterile PBS. Stepwise sucrose gradients were loaded by layering 20–30%, 30–40%, and 40–50% sucrose. The resuspended ultracentrifugation pellet obtained was loaded onto the gradients and centrifuged at 200,000× *g* for 4 h at 4 °C. Fractions collected from different sucrose layers were analyzed by Western blot to identify Cap-containing fractions. For sucrose exchange, Cap-positive sucrose fractions were diluted with 10 volumes of sterile PBS, followed by ultracentrifugation at 200,000× *g* for 4 h at 4 °C. The resulting pellet was gently resuspended in the buffer used for subsequent ion-exchange chromatography, consisting of sterile PBS adjusted to pH 7.8.

### 4.6. Purification of PCV3 Cap VLPs by Cation-Exchange Chromatography

The theoretical isoelectric point of PCV3 Cap was predicted using the ExPASy online tool (SIB Swiss Institute of Bioinformatics, Lausanne, Switzerland) to guide chromatographic purification. Because the purification buffer was PBS at pH 7.8, cation-exchange chromatography was selected for further purification of PCV3 Cap VLPs. Prior to column loading, sucrose-exchanged samples were clarified by centrifugation at 10,000× *g* for 10 min and filtered through a 0.22 μm membrane to remove large particulate impurities. Cation-exchange purification was performed using SP Sepharose™ Fast Flow resin (Cytiva, Marlborough, MA, USA). Bound proteins were eluted with NaCl-containing buffers at final concentrations of 400 mM and 1 M. Eluted fractions were concentrated by ultracentrifugation at 200,000× *g* for 4 h at 4 °C. Purified preparations were analyzed by SDS-PAGE and Western blot.

### 4.7. Transmission Electron Microscopy

PCV3 Cap VLP preparations purified by cation-exchange chromatography were submitted to the Instrumental Analysis Center of Shanghai Jiao Tong University (Shanghai, China) for transmission electron microscopy imaging.

### 4.8. Mouse Immunization

Six- to eight-week-old female BALB/c mice were randomly assigned into five groups: a blank control group (Control), a PBS plus adjuvant group, and three PCV3 Cap VLP immunization groups receiving low, medium, or high antigen doses of 10 μg, 20 μg, or 40 μg formulated with adjuvant. Each group contained 15 BALB/c mice aged 6–8 weeks. Immunization was performed by subcutaneous injection in the dorsal region using ISA 201 as the adjuvant. Antigen was diluted in sterile PBS to a final volume of 100 μL and emulsified with 100 μL ISA 201 immediately before injection. Mice were immunized three times at 2-week intervals.

### 4.9. Humoral Immune Response Analysis

Mouse sera were collected at 0, 7, 14, 21, 28, and 35 days after immunization for measurement of antigen-specific antibody responses. Serum IgG levels were determined using an indirect ELISA previously established in our laboratory. Plates were coated with pCold-SUMO-Cap protein expressed in *E. coli* as the capture antigen, and mice sera antibodies were detected using an HRP-conjugated anti-mouse secondary antibody. For IgG subclass analysis, sera collected at day 35 post-immunization were evaluated using the same indirect ELISA workflow, except that HRP-conjugated goat anti-mouse IgG1 and HRP-conjugated goat anti-mouse IgG2a antibodies were used as secondary antibodies to quantify IgG1 and IgG2a responses, respectively. In addition, the ability of post-immunization serum to recognize PCV3-related antigens was evaluated by indirect immunofluorescence assay using PK-15 cells persistently carrying PCV3 through serial passage, previously established in our laboratory, as target cells [32]. Cells were fixed with 4% paraformaldehyde and incubated with post-immunization serum as the primary antibody, followed by incubation with a fluorescence-conjugated secondary antibody. Nuclei were counterstained with DAPI, and fluorescence signals were observed using a fluorescence microscope. Serum from the control group was used in parallel as a negative control.

### 4.10. Cytokine mRNA Analysis in Splenic Lymphocytes After Immunization

Splenic lymphocytes were isolated from mice after immunization. Total RNA was extracted using a commercial Total RNA extraction kit (Thermo Fisher Scientific, Waltham, MA, USA) and reverse-transcribed into cDNA using a reverse transcription kit (Omega Bio-tek, Norcross, GA, USA) according to the manufacturers’ instructions. Relative mRNA expression levels of IFN-γ, TNF-α, IL-4, and IL-10 were quantified by SYBR Green-based RT-qPCR. Primer sequences are listed in Table 2.

### 4.11. Pig Immunization–Challenge Protection Assay

An immunization–challenge experiment was performed in 28-day-old PCV-negative female Duroc × Landrace × Yorkshire (DLY) pigs to evaluate the protective efficacy of PCV3 Cap VLPs in the natural host. A total of 15 pigs were randomly divided into three groups: negative control, challenge control, and PCV3 Cap VLP immunization, with five pigs per group. Pigs in the immunization group received 50 μg PCV3 Cap VLPs formulated with ISA adjuvant by intramuscular injection in the neck. At 28 days after immunization, pigs in the immunization and challenge control groups were challenged with PCV3, whereas pigs in the negative control group received an equivalent volume of sterile DMEM as mock challenge. The challenge was performed by intranasal and intramuscular inoculation using successfully rescued PCV3, at a dose of 5 × 10^11^ genome copies per pig, with 2 mL administered into each nostril and 1 mL injected intramuscularly into the right neck triangle area. PCV3 genome copies in serum were quantified using a fluorescent quantitative PCR kit (Beijing Scenk Biotechnology Development Co., Ltd., Beijing, China) according to the manufacturer’s instructions, and the PCV3 virus used for challenge had been prepared previously in our laboratory [32]. Serum samples were collected at 0, 3, 7, 14, 21 and 28 dpi, and the positive detection rate was recorded as the number of positive pigs/total pigs in each group.

### 4.12. Statistical Analysis

Statistical analyses were performed using GraphPad Prism 8 software. In the mouse immunization experiment, each group contained five biological replicates, and ELISA measurements were performed in technical replicates. IgG subclass and cytokine mRNA expression data were analyzed based on samples from individual mice. Comparisons among groups at a single time point were performed using one-way ANOVA, whereas longitudinal ELISA data were analyzed using two-way ANOVA. In the pig experiment, five pigs were included in each group, and serum viral load results are presented as positive detection rates. Because these data were primarily used to describe the trend of viral positivity after challenge, no further statistical comparison was performed. Statistical differences between the groups were defined as follows: * *p* < 0.05; ** *p* < 0.01. Data are presented as means ± SDs.

## Figures and Tables

**Table 1:** Positive detection rate of serum viral load in different groups after challenge.

Time After Challenge (dpi)	0	3	7	14	21	28
Negative control	0/5	0/5	0/5	0/5	0/5	0/5
Challenge control	0/5	5/5	5/5	5/5	5/5	5/5
PCV3 Cap VLPs	0/5	2/5	1/5	1/5	1/5	1/5

Data are presented as the number of positive pigs/total pigs.

**Table 2:** Sequences of primers.

Gene	Sequence (5′-3′)
*β-actin*	CGGTTCCGATGCCCTGAGGCTCTT
	CGTCACACTTCATGATGGAATTGA
*IFN-γ*	GGTCAACAACCCACAGGTCC
	CGAATCAGCAGCGACTCCTT
*TNF-α*	CAGGCGGTGCCTATGTCTC
	CGATCACCCCGAAGTTCAGTAG
*IL-4*	TCGGCATTTTGAACGAGGTC
	GAAAAGCCCGAAAGAGTCTC
*IL-10*	CAGAGCCACATGCTCCTAGA
	TGTCCAGCTGGTCCTTTGTT

## Data Availability

The original contributions presented in this study are included in the article. Further inquiries can be directed to the corresponding author.

## Abbreviations

The following abbreviations are used in this manuscript:

Cap	Capsid protein
VLPs	Virus-like particles
MBP	Maltose-binding protein
F2A	Foot-and-mouth disease virus 2A
BEVS	Baculovirus Expression Vector System
pH	polyhedrin
